# Molecular epidemiology of pneumococci obtained from Gambian children aged 2–29 months with invasive pneumococcal disease during a trial of a 9-valent pneumococcal conjugate vaccine

**DOI:** 10.1186/1471-2334-8-81

**Published:** 2008-06-11

**Authors:** Martin Antonio, Hannah Dada-Adegbola, Ekow Biney, Tim Awine, John O'Callaghan, Valentin Pfluger, Godwin Enwere, Brown Okoko, Claire Oluwalana, Adeola Vaughan, Syed MA Zaman, Gerd Pluschke, Brian M Greenwood, Felicity Cutts, Richard A Adegbola

**Affiliations:** 1 Medical Research Council Laboratories, Banjul, The Gambia; 2 Swiss Tropical Institute, Basel, Switzerland; 3 London School of Hygiene and Tropical Medicine, UK

## Abstract

**Background:**

The study describes the molecular epidemiology of *Streptococcus pneumoniae *causing invasive disease in Gambian children

**Methods:**

One hundred and thirty-two *S. pneumoniae *isolates were recovered from children aged 2–29 months during the course of a pneumococcal conjugate vaccine trial conducted in The Gambia of which 131 were characterized by serotyping, antibiotic susceptibility, BOX-PCR and MLST.

**Results:**

Twenty-nine different serotypes were identified; serotypes 14, 19A, 12F, 5, 23F, and 1 were common and accounted for 58.3% of all serotypes overall. MLST analysis showed 72 sequence types (STs) of which 46 are novel. eBURST analysis using the stringent 6/7 identical loci definition, grouped the isolates into 17 clonal complexes and 32 singletons. The population structure of the 8 serotype 1 isolates obtained from 4 vaccinated and 2 unvaccinated children were the same (ST 618) except that one (ST3336) of the isolates from an unvaccinated child had a novel ST which is a single locus variant of ST 618.

**Conclusion:**

We provide the first background data on the genetic structure of *S. pneumoniae *causing IPD prior to PC7V use in The Gambia. This data will be important for assessing the impact of PC7V in post-vaccine surveillance from The Gambia.

## Background

Pneumonia is a frequent cause of childhood deaths in many developing countries and its leading cause is *Streptococcus pneumoniae *[1-3]. In The Gambia, there is a high burden of pneumococcal disease in young children in both urban and rural settings [4-9]. During a recently concluded pneumococcal conjugate vaccine trial in The Gambia, the incidence of invasive pneumococcal disease in children who had not received the vaccine was 363 (95% CI 157, 715) per 100 000 person-years in children aged 6 weeks to 5 months and 576 (95%CI 369, 857) per 100,00 person years in those aged 6–11 months [7]. Similar figures have been reported from rural Kenya in a population that had not received Hib or pneumococcal conjugate vaccines [10]. The pneumococcus is also a major cause of otitis media, responsible for much morbidity in young children worldwide [11]. HIV infection increases susceptibility to pneumococcal infection and, as a consequence, the incidence of pneumococcal infection in children and adults is increasing in many parts of sub-Saharan Africa [12,13]. Emergence of antibiotic resistant pneumococci has complicated case-management strategies for pneumococcal disease. Therefore, long-term prevention of pneumococcal disease in young children by vaccination is a major international public health priority.

*S. pneumoniae *is subdivided into 91 different serotypes [14], all of which are capable of causing invasive disease. Geographic variations in the distribution of pneumococcal serotypes exist and some serotypes are associated selectively with disease in children or adults [15]. The advent of pneumococcal conjugate vaccines provides a potential way of preventing pneumococcal disease although available vaccines contain only a limited number of serotypes. The results of a recently concluded efficacy trial of a 9-valent pneumococcal conjugate vaccine (Wyeth) in Basse, a typical rural African setting in The Gambia, are highly encouraging as they showed protective efficacies, among children who received three doses of vaccine according to protocol, of 37% against radiological pneumonia, 77% against invasive pneumococcal disease of vaccine serotype, 50% against overall pneumococcal disease, 15% against all-cause hospitalizations and 16% against all-cause mortality [16]. The licensed 7-valent pneumococcal conjugate vaccine (Prevenar^®^) has had a dramatic impact on the incidence of invasive pneumococcal disease in vaccinated infants in the United States and has also protected unvaccinated adults through the induction of herd protection [17]. However, concerns have been raised about the long terms effects of vaccines containing limited numbers of serotypes on the overall pneumococcal population [17]. Thus, prior to the planned introduction of Prevenar^R ^into the routine immunisation programme of The Gambia, we studied 131 but presented data on 127 *S. pneumoniae *isolates obtained from vaccinated or unvaccinated children (<2 years old) during The Gambian vaccine trial (between August 2000 and April 2004) by BOX-PCR genotyping and MLST to determine the current population structure of pneumococci in the country.

## Methods

### Study population

Seven thousand, five-hundred and fifty-nine samples including blood (6964), CSF (242), lung aspirate (123) or pleural effusion (230) were obtained from 4,944 children investigated for possible invasive pneumococcal disease during the course of a 9-valent pneumococcal conjugate vaccine trial (PCV9) conducted in The Gambia [7,16]. When more than one isolate was obtained during a single episode of illness, for example from blood and CSF, multiple isolates have been included in the analysis only if they differed by MLST and/or antibiotic susceptibility pattern. Only one child had two episodes of illness. One pneumococcal isolate was not viable after freezing and has not been tested further. Consequently, 132 *S. pneumoniae *isolates were recovered from vaccinated and unvaccinated children aged 2–29 months during the course of a pneumococcal conjugate vaccine trial conducted in The Gambia of which 131 were characterized by serotyping, antibiotic susceptibility, BOX-PCR and MLST and 127 had unique MLST and/or antibiotic susceptibility pattern and were included in this study. Details of how children were enrolled, followed up and investigated have been described previously [7,16]. Written consent was obtained from the parents and the trial was approved by the Joint Gambia Government and MRC Ethics Committee and Ethics Committees at the London School of Hygiene & Tropical Medicine and the World Health Organization.

### Isolation and serotyping of *S. pneumoniae*

*S. pneumoniae *isolates were obtained from lung aspirate, blood, CSF, pus or pleural fluid using an automated culturing system and/or by direct plating onto solid blood agar plates at the patient's bedside as previously described [7,16]. Pneumococci were serotyped initially by latex agglutination in The Gambia; serotype identification was subsequently confirmed using capsular swelling (Quellung reaction) at the Health Protection Agency (HPA) laboratory in Colindale, UK, using commercially available antisera (Statens Serum Institute, Copenhagen, Denmark) as described previously [7,16].

### Antimicrobial susceptibility testing

Antimicrobial susceptibility was determined by measurement of the minimum inhibitory concentration (MIC). MIC of each isolate was tested by E-strips (AB Biodisk, Solna, Sweden) following the manufacturer's instructions. The E-strips used tested for penicillin G, chloramphenicol, ampicillin, tetracycline, cefotaxime and cotrimoxazole sensitivity. *S. pneumoniae *ATCC 49619 strain was used as a control and data were interpreted as susceptible, intermediate or resistant following the manufacturer's instructions.

The MRC microbiology laboratory submits to the external quality assurance programme of the United Kingdom National External Quality Assessment Service [18]

### BOX PCR genotyping

Pneumococcal strain typing by BOX-PCR was performed as described previously [19] with slight modifications. Briefly, a single pneumococcal colony from a blood plate instead of pure DNA was used as the template for PCR (40 cycles of 1 min at 94°C, 1 min at 55°C, and 1 min at 74°C). The amplified products were separated on a 1% agarose gel and banding patterns were visualized with ethidium bromide staining and UV illumination with a gel documentation system (Gel Doc 2000; Bio-Rad, UK).

### Multi locus sequencing typing (MLST)

*S. pneumoniae *isolates were streaked on blood agar and incubated at 37°C for 18 hours. A single colony from each isolate was picked, restreaked and incubated at 37°C for 18 hours. Genomic DNA templates were prepared from a loopful of bacteria as described in the manufacturer's instructions (Qiagen Genomic DNA Kit, UK). MLST was performed as described [20]. The seven genes targeted were *aroE, gdh, gki, recP, spi, xpt *and *ddl*. Amplifications for all genes were carried out with approximately 0.2 μg DNA template, 250 μM (each) deoxynucleoside triphosphates, 2.5 mM MgCl_2_, 25 pmol of primers, and 1 U of *Taq *polymerase (Qiagen.) in a 25 μl reaction mixture. PCR cycling conditions were a 10 min hold at 94°C, followed by 34 cycles of 94°C for 1 min, 55°C for 1 min, and 72°C for 1 min, and a final extension at 72°C for 5 min. Two μl of reaction mixtures were separated by 1.0% agarose gel electrophoresis and visualized with ethidium bromide staining and UV illumination with a gel documentation system (Gel Doc 2000; Bio-Rad, UK).

PCR products were sent to Macrogen (South Korea) for purification and DNA sequencing on both strands. Sequences were edited and complementary sense and antisense fragments were aligned using the Laser Gene DNA star 7.1 software. The sequences were submitted to the MLST database website [21] and assigned existing or novel allele type numbers and sequence type numbers defined by the database. This multi microorganism database defines a novel allele type if it contains one or more nucleotide changes from existing allele sequences. Composite sequence types (STs) are assigned based on the set of allele types derived from each of the seven loci. STs were analyzed for relatedness using the eBURST v3 program [22]. Cluster analysis of allelic profiles was performed using a categorical coefficient and a graphic method called a minimum spanning tree with Bionumerics software (version 4.0; Applied Maths, Sint-Martens-Latem, Belgium).

## Results

### Microbiology

All 127 invasive isolates were screened for antimicrobial susceptibility by disc diffusion and MIC was detected by the E-test technique. 100% of the isolates were susceptible to cefotaxime and ampicillin, whilst susceptibility to penicillin, chloramphenicol, cotrimoxazole, and tetracycline were 97.6%, 88.8%, 80.0% and 46.4% respectively. Two isolates were resistant to three antibiotics (chloramphenicol, cotrimoxazole, and tetracycline) whereas 13 isolates were resistant to two antibiotics (tetracycline and chloramphenicol or cotrimoxazole). One child had two episodes of pneumococcal disease caused by different isolates. The isolate involved in the first episode of illness was susceptible to all antibiotics tested whereas the isolate from the second episode was resistant to tetracycline and chloramphenicol.

### Serotypes, clones and vaccination status

Twenty-nine different serotypes of pneumococci were detected among 127 isolates (Table 1). Serotypes 14, 19A, 12F, 5, 23F and 1 (in descending order), were common and accounted for 58.3% of all serotypes overall. Among unvaccinated children, serotypes 14, 5, 23F, 12F, 19A and 9L (in descending order) were commonest. Overall, 59 (46.4%) of the 127 serotypes were covered by the conjugates in the PCV9 vaccine (Table 1).

**Table 1:** Distribution of invasive paediatric *S. pneumoniae *isolates by vaccine and non-vaccine serotypes between children randomized to receive vaccine and those randomized to receive placebo (intent-to-treat analysis).

**No. of isolates**			
**Serotype**	**Unvaccinated**	**Vaccinated**	**Total**

1	3	5	**8**
2	1	0	**1**
4	3	0	**3**
**5**	**10**	**1**	**11**
6A	3	3	**6**
6B	4	1	**5**
8	0	2	**2**
9L	5	0	**5**
9V	2	2	**4**
12B	0	1	**1**
12F	7	5	**12**
14	17	3	**20**
16F	4	0	**4**
18C	1	0	**1**
18F	1	0	**1**
19A	6	6	**12**
19F	2	0	**2**
20	0	1	**1**
21	0	2	**2**
22A	1	1	**2**
23F	9	2	**11**
29	0	1	**1**
33F	0	2	**2**
35A	0	2	**2**
35B	0	1	**1**
38	2	0	**2**
40	0	1	**1**
45	1	0	**1**
46	0	3	**3**
**Total**	**82**	**45**	**127**

**Figure 1:**
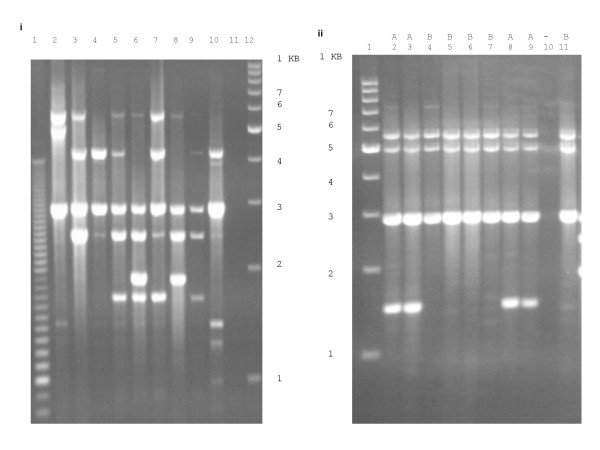
**BOX-PCR fingerprint patterns of invasive paediatric *S. pneumoniae *isolates collected during the PVT trial**. **(a) **PCR fingerprint patterns generated with BOX primer. Lanes 2–10 contained serotypes found in the 9-valent vaccine (1, 4, 5, 6B, 9V, 14, 18C, 19F & 23F) respectively. Lanes 1 &12 contained a molecular weight standard; 100-bp and 1 kb respectively. Lane 11; negative (no DNA) control. **(b) **BOX-PCR fingerprint patterns of invasive pneumococcal serotype 1 collected during the PVT trial. Lanes 2–9 &11 contained serotypes 1 isolates. Lane 1 contained a 1 kb molecular weight standard and lane 10 a negative (no DNA) control. There are two BOX-PCR fingerprint profiles designated A and B.

The genetic structure of the 127 isolates was examined using MLST and BOX-PCR analysis. BOX analysis revealed that all the 127 isolates shared an identical or closely related profile (representative isolates depicted in figure 1) and that this analysis can differentiate between serotypes (figure 1a) and within serotypes (figures 1b). For example we observed two fingerprinting patterns (A and B) for serotype 1 isolates (figure 1b)

**Figure 2:**
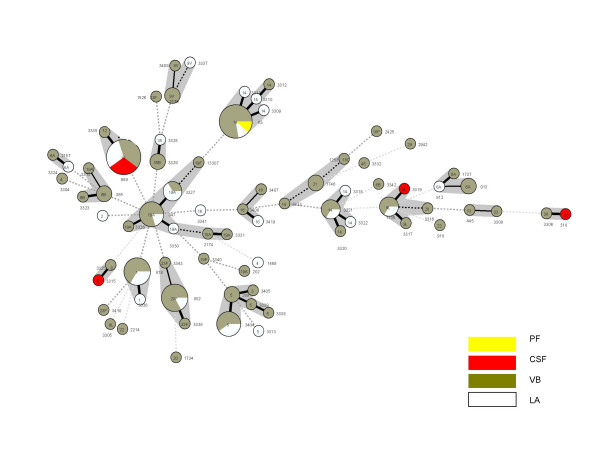
**Clustering of STs by use of the minimum spanning tree**. Each circle represents an ST, and the serotype number is indicated in the circle. The area of each circle corresponds to the number of isolates. Thick, short, solid lines connect single-locus variants and thin, longer, solid lines connect double-locus variants. Each colour represents the source of isolates. **Source**: LA (lung aspirate), CSF (cerebrospinal fluid), VB (venous blood), and PE (pleural effusion).

MLST showed that there were 72 sequence types (ST) (Table 2), 46 (64%) of which are novel, *i.e*. not found in the *S. pneumoniae *MLST database [21]. eBURST analysis using the stringent 6/7 identical loci definition, grouped these ST into 17 clonal complexes and 31 singletons indicating a large genetic diversity between the isolates (figure 2). The results of cluster analysis of all allelic profiles using the minimum spanning tree are shown in figure 2. In general, STs were restricted to the same serotype (Table 2) and the results between our data set and the pneumococcal MLST database [21] were concordant.

**Table 2:** Molecular characterisation of paediatric *S. pneumoniae *serotypes by MLST

		**Allelic profile**						
**Serotype**	**ST**	***aroE***	***gdh***	***gki***	***recP***	***spi***	***xpt***	***ddl***

Serotype 1	618	13	8	4	1	7	19	14
	3336	13	8	4	1	7	19	161
Serotype 2	3314	2	13	4	1	6	6	6
Serotype 4	1469	8	88	103	18	46	142	161
	3304	1	5	1	1	15	1	14
Serotype 5	289	16	12	9	1	41	33	33
	3338	16	12	9	1	4	33	9
	3404	16	12	9	1	176	33	33
	3313	2	12	9	10	17	1	33
	3339	16	12	9	1	41	33	9
Serotype 6A	912	6	57	34	28	7	1	9
	913	6	57	83	28	7	19	9
	1737	6	57	34	28	6	1	9
	2157	1	8	1	5	42	60	14
	3324	7	8	1	5	42	60	14
Serotype 6B	385	7	6	1	2	6	1	14
	3342	60	82	4	5	42	122	28
	3323	7	6	1	35	6	1	14
Serotype 8	3325	7	9	15	11	93	58	70
	3406	7	9	15	11	178	58	70
Serotype 9L	1735	6	5	2	5	36	1	5
	3305	1	8	4	15	16	3	31
	3317	6	5	2	6	36	1	5
Serotype 9V	2179	15	17	4	4	6	1	17
	3337	15	17	4	16	6	20	161
	3409	15	17	4	4	179	20	17
Serotype 12F	989	12	5	89	8	6	112	14
Serotype 12B	3335	12	5	89	8	6	25	14
Serotype 14	63	2	5	36	12	17	21	14
	3320	6	60	1	5	27	20	6
	3312	2	5	89	12	27	21	14
	3321	6	60	4	5	27	20	6
	3333	10	60	4	12	15	112	6
	3310	2	5	36	12	27	21	14
	3318	6	9	4	5	27	20	6
	3322	6	60	4	5	27	38	6
	3309	2	5	36	12	17	38	14
	3311	2	5	89	12	17	21	14
Serotype 16F	3341	23	12	4	1	6	12	74
	3407	23	189	4	12	43	4	74
	3408	23	189	4	12	15	241	74
	3419	6	189	4	12	15	241	74
Serotype 18C	1233	10	11	34	16	15	1	145
Serotype 18F	2425	10	5	34	16	36	142	31
Serotype 19A	202	8	16	19	15	6	40	26
	847	7	11	4	1	6	112	14
	2174	7	16	8	8	6	142	14
	3327	7	11	4	10	6	112	14
	3326	7	11	4	1	6	112	15
	3330	7	16	4	1	6	112	14
	3315	5	6	1	2	6	1	5
	3331	7	16	8	8	4	142	14
Serotype 19F	3307	2	5	4	10	6	1	14
	3340	16	16	19	1	6	20	33
Serotype 20	1734	5	31	8	1	27	1	31
	3316	6	5	2	5	67	49	18
Serotype 21	1746	10	13	4	16	15	1	6
Serotype 22	910	5	5	6	5	9	17	19
	2214	5	5	4	4	11	19	18
Serotype 23F	802	10	13	53	1	72	38	31
	1526	7	13	8	6	25	1	8
	3343	7	13	53	1	72	38	14
	3410	118	8	4	12	72	19	5
Serotype 29	2942	12	111	34	84	19	83	6
Serotype 33	445	2	5	29	5	42	3	18
	3308	2	5	29	18	13	3	18
Serotype 35B	3329	7	13	4	8	6	20	18
	3328	7	13	4	8	6	1	18
Serotype 38	310	1	43	41	18	13	49	6
	3306	1	43	41	18	13	49	5
Serotype 45	3332	8	15	89	1	19	1	6
Serotype 46	3334	12	5	53	8	7	27	14

**Figure 3:**
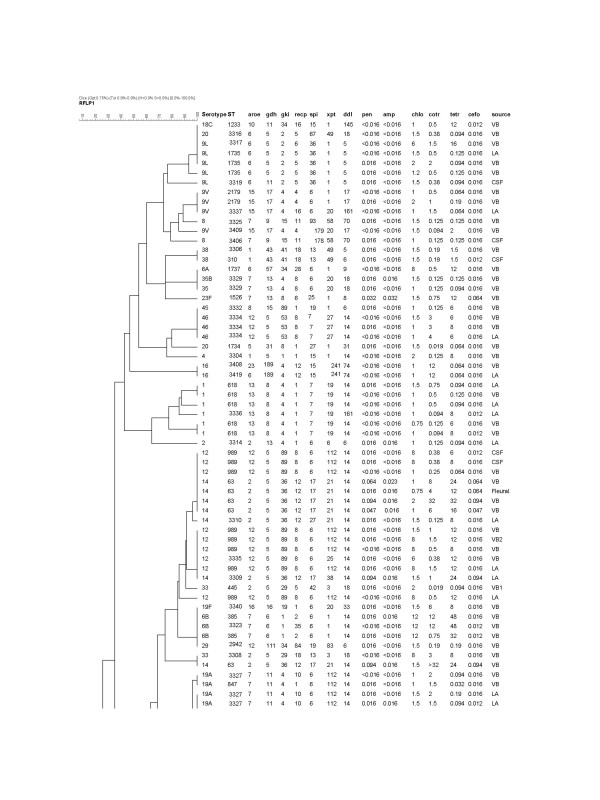
**Dendrogram showing the relatedness among the STs and serotypes of *S. pneumoniae *by use of UPGMA from BOX-PCR**. **Notes: Antimicrobial patterns**: pen (penicillin G); amp (ampicillin), chlo (chloramphenicol), cotr (cotrimoxazole tetr (tetracycline) and cefo (cefotaxime); **Source**: LA (lung aspirate), CSF (cerebrospinal fluid), VB (venous blood), and PE (pleural effusion).

**Figure 4:**
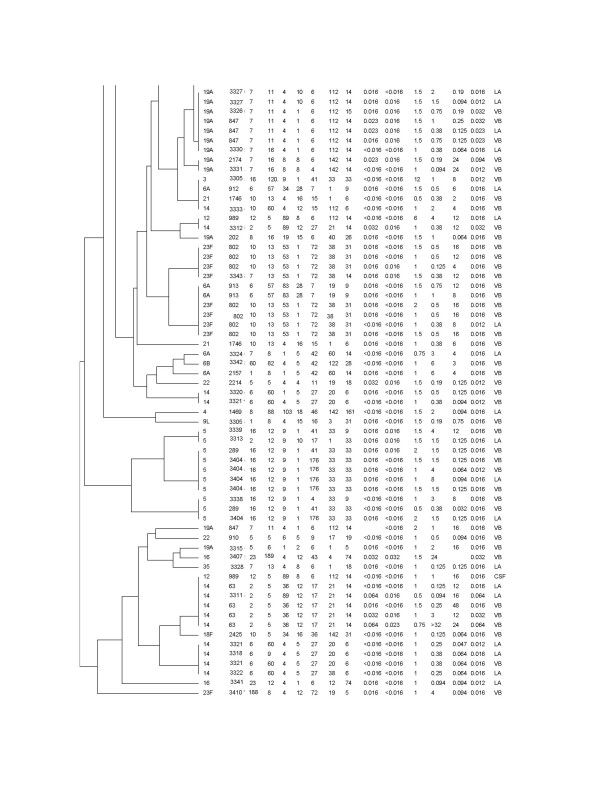
**Dendrogram showing the relatedness among the STs and serotypes of *S. pneumoniae *by use of UPGMA from BOX-PCR**. **Notes: Antimicrobial patterns**: pen (penicillin G); amp (ampicillin), chlo (chloramphenicol), cotr (cotrimoxazole tetr (tetracycline) and cefo (cefotaxime); **Source**: LA (lung aspirate), CSF (cerebrospinal fluid), VB (venous blood), and PE (pleural effusion).

A complete data set for BOX-PCR dendrogram, MLST, serotyping, antibiotic susceptibility pattern and the source of pneumococcal isolates used in this study are shown in the figures 3 and 4'.

### New alleles obtained in this study

We discovered seven new alleles including one *aroE *(118), two *gdh *(188, 189), three *spi *(176, 178,179) and one *xpt *(241).

## Discussion

One hundred and thirty-two *S. pneumoniae *isolates were recovered from vaccinated and unvaccinated children aged 2–29 months during the course of a pneumococcal conjugate vaccine trial conducted in The Gambia of which 131 were characterized by serotyping, antibiotic susceptibility, BOX-PCR and MLST and 127 had unique MLST and/or antibiotic susceptibility pattern and were included in this study.

There was no particular association of clones with specific presentations of invasive pneumococcal disease (data not shown) although three out of six (50%) cases of meningitis were caused by pneumococci belonging to serotype 12F with ST989. This result concurs with findings in Northern Ghana [23] and Niger where serotype 12F ST989 isolates have been detected in patients with meningitis. Serotype 12F ST989 isolates have also been identified in nasopharyngeal carriers in a village in Kenya [24] and in Qatar [25]. Similarly, a SLV of ST989, serotype 12B ST2211, was responsible for a case of meningitis in Niger [25].

In this study, we found twenty-nine different serotypes corresponding to seventy-two MLST types (sequence types). MLST is the definitive, internationally accepted method for monitoring the spread of clones through the pneumococcal population [20,21,26]. Our data showed that serotype and genotype distribution varied considerably in vaccinated and unvaccinated children suggesting a high degree of fluidity in the pneumococcal population dynamics (figure 2).

Although the 9-valent vaccine used in our trial contained glycoproteins for serotypes 1 and 9V, pneumococci of these serotypes caused a small number of cases of invasive disease with no reduction in cases in vaccinated children. For example, 4 vaccinated children (from whom 5 isolates were obtained – Table 1) had invasive disease due to a serotype 1 infection compared with 2 unvaccinated children (from whom 3 isolates were obtained – Table 1). There was no clustering of cases of serotype 1 in either vaccinated or unvaccinated groups over time during the period 2002–2004. The very small number of serotype 1 and 9V cases detected precludes precise estimation of serotype-specific efficacy for these two serotypes but these findings raise the possibility that efficacy was lower against serotypes 1 and 9V than against other vaccine-type serotypes, despite the fact that the vaccine elicited strong immune responses, as measured by ELISA, for all serotypes contained in the 9-valent vaccine, including serotypes 1 and 9V (data not shown). All but one of the seven serotype 1 isolates in our study had the same genotype, ST 618. One of the isolates, from an unvaccinated child, had a novel ST (ST3336) which is a SLV of ST 618. ST 618 and its single and double locus variant have recently been observed to cause meningitis in Ghana, Burkina Faso and Egypt [25]. Furthermore, ST618 belongs to the same clonal complex (ST 217) of serotype 1 that has caused an outbreak of meningitis in Northern Ghana [23], indicating a dominant West African clone. The ST217 hypervirulent clonal complex is typical of lineage B serotype 1 pneumococci that are predominant within Africa and Israel [26]. A lower efficacy for serotype 1 and 9V could be explained by heterogeneity or intraspecies variations among these invasive pneumococci.

Isolates belonging to the same genotype (clone) can express different types of capsular polysaccharide through horizontal recombination of pneumococcal DNA by natural transformation [20]. This phenomenon, capsular switching, leads to the emergence of a new clone expressing a different capsule [17]. There was no direct evidence of capsular switching within our isolates. However, comparative analysis of our serotype and MLST results with those published in the MLST database showed that some genotypes found in our isolates express different serotypes in other areas. For example, we found genotype ST63 to be serotype 14 and associated with bacteremia and pneumonia (figure 2) and ST63 serotype 14 is associated with meningitis in Niger [27]. Elsewhere, ST63 has been found to express serotype 15A, 19A and 23F capsular polysaccharides [25], suggesting that ST63 has a high propensity for capsular switching. ST912 expressing serotype 9V capsular polysaccharide has previously been isolated from a carrier in The Gambia [25] but in this study we found ST912 expressing serotype 6A polysaccharide. Similarly, ST2179 expressing serotype 6A capsular polysaccharide has previously been isolated from a Gambian carrier [25] while in this study we found ST2179 expressing serotype 9V polysaccharide and causing invasive disease, suggesting capsular switching. Similarly, ST2174 expressing a serotype 23F capsule has previously been isolated from a carrier in The Gambia [25] but in this study we found ST2174 expressing serotype 19A capsular polysaccharide again suggesting capsular switching. This is plausible as cluster analysis from our data shows that serotype 19A and 23F are genetically linked (figure 2). Although our data is suggestive that capsular switching can occur naturally, a large dataset will be required to show the impact of conjugate vaccination on this process. We have recently set up nasopharyngeal pneumococcal carriage studies in 21 villages in The Gambia to address this question [28].

The use of PCV has the potential to reduce substantially the number of drug-resistant pneumococcal infections [29]. Antimicrobial resistance in pneumococci is closely linked to serotype and genotype. Although we found no penicillin resistant isolates in this study, there is evidence from The Gambia that isolates of vaccine serotype have significantly higher intermediate resistance to penicillin than those of non-vaccine serotype (p < 0.01) [28]. Similarly, isolates of 9-valent serotype were less likely to be resistant to tetracycline than those of a non-vaccine serotype (39.0% vs. 52.6%; p < 0.05). In contrast, isolates of non-vaccine serotype were more likely to be resistant to chloramphenicol (p < 0.0001). Interestingly, we identified two serotype 6B isolates with ST385 and ST3323 which were-resistant to three antibiotics; chloramphenicol, cotrimoxazole and tetracycline. ST385 and ST3323 are also SLV and DLV respectively to ST273. ST273 is known to be a major pandemic antimicrobial-resistant clone, Greece 6B-22 [30,31] of pneumococci as defined by the pneumococcal molecular epidemiology network.

## Conclusion

Plans are being developed for the introduction of pneumococcal conjugate vaccines into the routine immunization programmes of several countries in Africa. We conclude that in order to assess the impact of this intervention on the prevalence of antimicrobial resistance in strains of *S. pneumoniae *causing IPD and on the propensity of PCV to cause serotype replacement it will be necessary to monitor the antimicrobial susceptibility of *S. pneumoniae *isolates from cases of IPD in parallel with serotypic and genotypic characterization before and after the introduction of these programmes.

## Competing interests

The authors declare that they have no competing interests.

## Authors' contributions

MA and RA conceived the study and wrote the paper with GE, FC and BMG. MA, HD-A, JO'C and VP performed PCR, sequence reactions, edited and aligned DNA sequences for MLST analysis. EB cultured and identified bacteria isolates from clinical samples. GE, BO, CO, AV, SMAZ and FC participated in field and clinical aspects of the study. TA was involved in statistical analysis. All authors read and approved the final manuscript.

## Pre-publication history

The pre-publication history for this paper can be accessed here:


